# Prognostic significance of the Controlling Nutritional Status (CONUT) score in predicting postoperative complications in patients with Crohn’s disease

**DOI:** 10.1038/s41598-020-76115-0

**Published:** 2020-11-04

**Authors:** Xue Dong, Shasha Tang, Wei Liu, Weilin Qi, Linna Ye, Xiaoyan Yang, Xiaolong Ge, Wei Zhou

**Affiliations:** 1 Department of General Surgery, Sir Run Run Shaw Hospital, School of Medicine, Zhejiang University, 3 East Qingchun Road, Hangzhou, 310016 Zhejiang China; 2 Department of Radiology, Sir Run Run Shaw Hospital, School of Medicine, Zhejiang University, Hangzhou, 310016 Zhejiang China; 3 Department of Gastroenterology, Sir Run Run Shaw Hospital, School of Medicine, Zhejiang University, Hangzhou, 310016 Zhejiang China

**Keywords:** Diseases, Gastroenterology, Risk factors

## Abstract

Preoperative immune-nutritional status is correlated with postoperative outcomes. The Controlling Nutritional Status (CONUT) score is a useful tool for predicting the postoperative outcomes of cancer surgery. This study aimed to evaluate whether the CONUT score could predict postoperative complications in Crohn’s disease (CD) patients. In total, 202 CD patients were eligible. Univariate and multivariate analyses were performed to identify risk factors for postoperative complications. Receiver operating characteristic (ROC) curves were generated to examine the cutoff value for predictors of postoperative complications. Among all the patients, 66 developed postoperative complications. The cut-off value of the CONUT score was 3.5 for complications. Eighty-one patients had a low CONUT score (< 3.5), and 121 patients had a high CONUT score (> 3.5). There was a significant difference in postoperative complications between the groups with low and high CONUT score (17.3% vs. 43.0%, p < 0.001). Patients with high CONUT score had low body mass index (BMI), more mild postoperative complications (p = 0.001) and a longer postoperative stay (p = 0.002). Postoperative complications were correlated with BMI, preoperative albumin, the preoperative CONUT score, and preoperative infliximab use. Then, the preoperative CONUT score was an independent risk factor for complications (OR 3.507, 95% CI 1.522–8.079, p = 0.003). ROC analysis showed that the CONUT score was a better predictor of postoperative complications in CD patients than albumin and the prognostic nutritional index. Thus, a preoperative CONUT score cut-off value of more than 3.5 could help to identify patients with a high possibility of malnutrition and postoperative complications.

## Introduction

Crohn’s disease (CD) is a transmural inflammatory disease of the intestinal mucosa, and it occurs discontinuously in any segment of the digestive tract^1^. The interplay between genetic factors, environmental factors, and gut microbiota is reported to be involved in the pathogenesis of Crohn’s disease; this interplay leads to an abnormal mucosal immune response and an imbalanced intestinal epithelial barrier^2,3^. Almost 80% of CD patients are reported to receive surgical treatment due to the failure of medical therapy during their lifetime^4^. Postoperative complications, especially infectious complications, are common in CD patients who receive digestive surgery and occur at a rate of 10% to 37%^5^. Therefore, reliable clinical predictors of postoperative complications are needed in CD.

The nutritional status and immunological status of CD patients are important factors for postoperative complications due to the pathophysiological characteristics of CD. Thus, the roles of albumin (ALB), body mass index (BMI), C-reactive protein (CRP), prognostic nutritional index (PNI), faecal calprotectin, subcutaneous fat area (SFA), interleukin-6 and other indicators are studied in predicting postoperative complications in CD^6–10^. For example, Dreznik et al.^11^ suggested that the nutritional status of CD patients needs to be optimized to avoid hazardous surgical complications, and postoperative complications in patients with low albumin can be minimized by nutritional support. Grass et al.^12^ reported that malnutrition was a major risk factor for postoperative complications, and postoperative morbidity can be decreased efficiently by enteral and parenteral routes. Additionally, a high preoperative CDAI score with severe inflammatory response could predict negative postoperative outcomes^13^. Therefore, it is necessary to find a scoring system to assess both nutritional status and immune status.

The Controlling Nutritional Status (CONUT) score is calculated based on the serum albumin level, total blood cholesterol concentration, and total peripheral lymphocyte count, and this objective tool was first reported for the early detection of hospital malnutrition in 2005^14^. The CONUT system is considered to be associated with the host’s nutritional and immune status based on these three laboratory parameters^15^. The CONUT is widely used as a prognostic indicator in cancer patients. Kuroda et al.^16^ reported that the CONUT score tended to estimate nutritional status and predict long-term overall survival in gastric cancer patients after surgery. The early postoperative CONUT score was found to be an independent risk factor for postoperative complications in patients with hepatocellular carcinoma after liver resection^17^. Recently, a meta-analysis showed that the CONUT score was helpful for predicting which patients with gastrointestinal and hepatopancreatobiliary cancers are at an increased risk of mortality and postoperative complications^18^. However, the clinical significance of the CONUT system for predicting postoperative outcomes in CD remains unknown.

This study analysed a large cohort of CD patients in the Inflammatory Bowel Disease Center of our hospital to demonstrate the role of the CONUT score in CD patients. The aim of the current study was to evaluate the relationship between the CONUT score and postoperative complications and to find a reliable and simple indicator to reflect immune-nutritional status in CD.

## Results

### Study population and baseline characteristics

A total of 202 CD patients were enrolled in the study; 135 (66.8%) were male, and the mean age of the patients was 36.5 ± 0.9 years. In total, 202 patients received an anastomosis after bowel resection, and 60 patients received a protective ostomy. The mean disease duration before surgery was 48.7 ± 3.4 months. Ileocolonic involvement was the most common disease pattern. Preoperatively, 39 (19.3%) patients had a medical history of azathioprine, 24 (11.9%) of infliximab, 55 (27.2%) of 5-ASA, 10 (5.0%) of corticosteroids, and 70 (36.1%) of enteral nutrition. The Montreal classification and the history of medical treatment are shown in Table 1. The mean CONUT score was 4.3 ± 0.2, and the mean PNI score was 41.7 ± 0.5. The ROC curve analysis showed that the CONUT cut-off value was 3.5. Thus, 81 (40.1%) patients were in the low CONUT group, with a score less than 3.5, and 121 (59.9%) were in the high CONUT group, with a score greater than 3.5.

**Table 1:** Baseline characteristics of all the patients.

Characteristics	All (202)	Low CONUTS (81)	High CONUTS (121)	P value
Age^a^, year	36.5 ± 0.9	35.6 ± 1.3	37.0 ± 1.2	0.429
Men	135 (66.8)	58 (71.6)	77 (63.6)	0.238
BMI^a^, kg/m^2^	18.7 ± 0.2	19.3 ± 0.3	18.3 ± 0.2	0.009
**Comorbidities**				
Diabetes mellitus	10 (5.0)	4 (4.9)	6 (5.0)	0.995
Hypertension	11 (5.4)	4 (4.9)	7 (5.8)	0.794
Preoperative hemoglobin^a^	11.8 ± 0.1	12.6 ± 0.2	11.4 ± 0.2	< 0.001
Preoperative albumin^a^	36.1 ± 0.4	40.0 ± 0.4	33.5 ± 0.5	< 0.001
Preoperative C-reactive protein^a^	13.2 ± 2.0	8.4 ± 1.1	16.4 ± 3.2	0.048
Preoperative erythrocyte sedimentation rate^a^	16.7 ± 1.1	14.0 ± 1.4	18.6 ± 1.5	0.033
Preoperative white blood cell^a^	5.9 ± 0.2	5.9 ± 0.2	5.9 ± 0.3	0.923
Preoperative red blood cell^a^	4.23 ± 0.04	4.47 ± 0.05	4.08 ± 0.06	< 0.001
Mean disease duration before surgery^a^, month	48.7 ± 3.4	40.4 ± 5.0	54.2 ± 4.4	0.043
**Montreal classification**				
Age, years				
A1 (≤ 16)	2 (1.0)	1 (1.2)	1 (0.8)	0.642
A2 (17–40)	136 (67.3)	55 (67.9)	81 (66.9)	0.887
A3 (> 40)	64 (31.7)	25 (30.9)	39 (32.2)	0.838
Location				
L1 (ileal)	76 (37.6)	26 (32.1)	50 (41.3)	0.185
L2 (colonic)	19 (9.4)	8 (9.9)	11 (9.1)	0.851
L3 (ileocolonic)	95 (47.0)	44 (54.3)	51 (42.1)	0.089
L4 (upper gastrointestinal)	26 (12.9)	9 (11.1)	17 (14.0)	0.541
Behavior				
B1 (inflammatory/failure of medical therapy)	10 (5.0)	2 (2.5)	8 (6.6)	0.164
B2 (stricturing)	143 (70.8)	55 (67.9)	88 (72.7)	0.460
B3 (penetrating)	66 (32.7)	29 (35.8)	37 (30.6)	0.438
Perianal disease	59 (29.2)	24 (29.6)	35 (28.9)	0.914
Operative time^a^, min	191.1 ± 4.2	182.0 ± 6.9	197.3 ± 5.1	0.071
First time operated	131 (64.9)	54 (66.7)	77 (63.6)	0.658
Laparoscopic surgery	120 (59.4)	60 (74.1)	60 (49.6)	0.001
Conversion	40 (19.8)	11 (13.6)	29 (24.0)	0.069
**Preoperative treatment**				
Azathioprine	39 (19.3)	17 (21.0)	22 (18.2)	0.620
Infliximab	24 (11.9)	8 (9.9)	16 (13.2)	0.471
5-ASA	55 (27.2)	21 (25.9)	34 (28.1)	0.734
Corticosteroids	10 (5.0)	3 (3.7)	7 (5.8)	0.497
Enteral nutrition	70 (36.1)	29 (35.8)	41 (33.9)	0.779
Others	14 (6.9)	3 (3.7)	11 (9.1)	0.140

Values in parentheses are percentages unless indicated otherwise.

BMI, body mass index; CONUTS, controlling nutritional status score.

^a^Values are mean ± SE.

### Operative data and postoperative complications

For 131 (64.9%) patients, this was their first surgery. A total of 120 (59.4%) patients underwent laparoscopic surgery successfully; however, 40 (19.8%) experienced conversion to laparotomy. The mean operative time was 191.1 ± 4.2 min. The low CONUT group had a higher rate of laparoscopic surgery than the high CONUT group (74.1% vs. 49.6%, p = 0.001), which might be associated with nutrition status, disease activity, and surgical history. After surgery, a total of 136 (67.3%) CD patients recovered uneventfully, and 66 (32.7%) patients had postoperative complications: 41 (20.3%) had mild complications, and 34 (16.8%) had major complications. The common complications in our hospital were wound infection (10.4%), early postoperative bowel obstruction (6.4%), and gastrointestinal bleeding (5.9%). The incidence of intra-abdominal abscess and anastomotic leakage was only 2.0% and 3.0% in our centre, and there was no significant difference between the low and high CONUT groups. The mean postoperative stay was 10.1 ± 0.4 days. In the low CONUT group, 14 (17.3%) patients had postoperative complications, which was significantly lower than the number in the high CONUT group (17.3% vs. 43.0%, p < 0.001). Therefore, the mean postoperative stay was also longer in the high CONUT group (8.6 ± 0.6 vs. 11.1 ± 0.5, p = 0.002). More details are shown in Table 2.

**Table 2:** Comparison of postoperative complications between CD patients with high- and low preoperative CONUTS.

Characteristics	All (n = 202)	Low CONUTS (81)	High CONUTS (121)	P value
**Postoperative complications**	66 (32.7)	14 (17.3)	52 (43.0)	< 0.001
Mild complications (grade I to II)	41 (20.3)	8 (9.9)	33 (27.3)	0.003
Wound infection	21 (10.4)	4 (4.9)	17 (14.0)	–
Early postoperative bowel obstruction	13 (6.4)	2 (2.5)	11 (9.1)	–
Postoperative blood transfusions	5 (2.5)	2 (2.5)	3 (2.5)	–
Line sepsis	2 (1.0)	0 (0)	2 (1.7)	–
Major complications (grade III to IV)	34 (16.8)	9 (11.1)	25 (20.7)	0.075
Gastrointestinal bleeding	12 (5.9)	4 (4.9)	8 (6.6)	–
Anastomotic leakage	6 (3.0)	3 (3.7)	3 (2.5)	–
Abdominopelvic collection	4 (2.0)	0 (0)	4 (3.3)	–
Pleural effusion	1 (0.5)	0 (0)	1 (0.8)	–
Intra-abdominal abscess	4 (2.0)	1 (1.2)	3 (2.5)	–
Stoma complications	4 (2.0)	1 (1.2)	3 (2.5)	–
Septic shock	1 (0.9)	0 (0)	1 (0.8)	–
Sepsis	2 (1.0)	0 (0)	2 (1.7)	–
Grade V	0 (0)	0 (0)	0 (0)	–
**Postoperative stay**^**a**^,** days**	10.1 ± 0.4	8.6 ± 0.6	11.1 ± 0.5	0.002

Values in parentheses are percentages unless indicated otherwise.

^a^Values are mean ± SE.

### Factors associated with postoperative complications

Univariate and multivariate analyses were performed to identify risk factors for complications in CD patients. The univariate analysis showed that BMI, preoperative albumin, the CONUT score, and preoperative use of infliximab were associated with postoperative complications. Moreover, there was no significant difference in PNI between the group with and without complications (42.3 ± 0.5 vs. 40.4 ± 0.9, p = 0.079). Then, the multivariate logistic regression analysis identified the preoperative CONUT score and infliximab as independent risk factors associated with postoperative complications (OR = 3.507, 95% CI 1.522–8.079, P = 0.003; OR = 2.619, 95% CI 1.050–6.531, P = 0.039) (Tables 3 and 4).

**Table 3:** Univariate analysis of risk factors associated with postoperative complications.

Characteristics	Without complications (136)	With complications (66)	P value
Men	91 (66.9)	44 (66.7)	0.972
BMI^a^, kg/m^2^	19.1 ± 0.2	18.1 ± 0.3	0.011
**Comorbidities**			
Diabetes mellitus	7 (5.1)	3 (4.5)	0.852
Hypertension	7 (5.1)	4 (6.1)	0.790
Preoperative hemoglobin^a^	12.0 ± 0.2	11.5 ± 0.2	0.092
Preoperative albumin^a^	36.7 ± 0.5	34.8 ± 0.8	0.021
Preoperative C-reactive protein^a^	10.7 ± 1.5	18.3 ± 5.2	0.074
Preoperative erythrocyte sedimentation rate^a^	16.5 ± 1.2	17.2 ± 2.0	0.768
Preoperative white blood cell^a^	5.66 ± 0.20	6.46 ± 0.35	0.057
Preoperative red blood cell^a^	4.27 ± 0.05	4.16 ± 0.08	0.238
Preoperative CONUTS	4.0 ± 0.2	5.1 ± 0.3	0.002
Mean disease duration before surgery^a^, month	48.0 ± 4.2	50.2 ± 5.5	0.758
**Montreal classification**			
Age, years			
A1 (≤ 16)	1 (0.7)	1 (1.5)	0.548
A2 (17–40)	87 (64.0)	49 (74.2)	0.144
A3 (> 40)	48 (35.3)	16 (24.2)	0.113
Location			
L1 (ileal)	48 (35.3)	28 (42.4)	0.327
L2 (colonic)	10 (7.4)	9 (13.6)	0.151
L3 (ileocolonic)	67 (49.3)	28 (42.4)	0.361
L4 (upper gastrointestinal)	18 (13.2)	8 (12.1)	0.824
Behavior			
B1 (inflammatory/failure of medical therapy)	8 (5.9)	2 (3.0)	0.361
B2 (stricturing)	95 (69.9)	48 (72.7)	0.673
B3 (penetrating)	47 (34.6)	19 (28.8)	0.412
Perianal disease	35 (25.7)	24 (36.4)	0.119
Operative time^a^, min	186.4 ± 4.9	200.9 ± 7.5	0.101
First time operated	94 (69.1)	37 (56.1)	0.068
Laparoscopic surgery	86 (63.2)	34 (51.5)	0.112
Conversion	28 (20.6)	12 (18.2)	0.687
**Preoperative treatment**			
Azathioprine	31 (22.8)	8 (12.1)	0.071
Infliximab	11 (8.1)	13 (19.7)	0.017
5-ASA	32 (23.5)	23 (34.8)	0.090
Corticosteroids	6 (4.4)	4 (6.1)	0.618
Enteral nutrition	52 (38.2)	18 (27.3)	0.125
Others	11 (8.1)	3 (4.5)	0.336

Values in parentheses are percentages unless indicated otherwise.

CONUTS, controlling nutritional status score; BMI, body mass index.

^a^Values are mean ± SE.

**Table 4:** Multivariate analysis of factors associated with postoperative complications.

Characteristics	Multivariate		
	P value	OR	95% CI
BMI	0.070	1.797	0.954–3.388
Preoperative albumin	0.878	0.943	0.445–1.999
Preoperative CONUTS	0.003	3.507	1.522–8.079
Infliximab	0.039	2.619	1.050–6.531

BMI, body mass index; CONUTS, controlling nutritional status score.

### Predictive accuracy of the CONUT score vs. other scoring systems for postoperative complications

**Figure 1:**
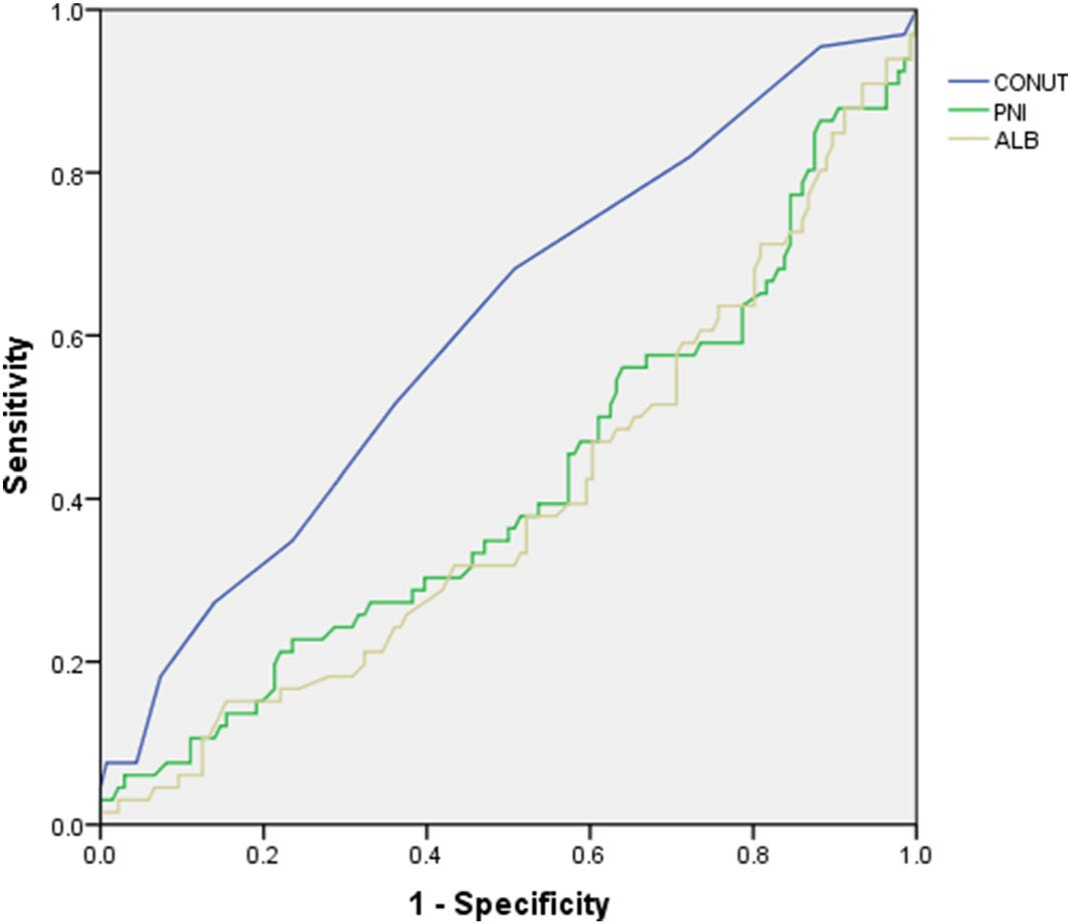
ROC curve for preoperative CONUTS, PNI, ALB predictive of postoperative complications. ROC, receiver operating characteristic; CONUTS, controlling nutritional status score; PNI, prognostic nutritional index; ALB, albumin.

It has been reported that ALB and PNI constitute a practical predictive index for postoperative complications in patients with CD or other digestive diseases^8,10^. ROC curve analysis was performed to examine the predictive accuracy of the CONUT score, ALB, and PNI. The areas under the curve (AUC) for the CONUT score, ALB, and PNI were 0.611, 0.399, and 0.418, respectively, which indicated that the CONUT score might be a better predictor of postoperative complications (Fig. 1). Additionally, the sensitivity of the CONUT score was 78.8%, and the specificity was 50.7%. The Youden index of the CONUT score was 0.281.

## Discussion

In patients with CD, malnutrition and systemic inflammation are the main risk factors for postoperative complications. In the current study, our results linked high CONUT scores in CD patients undergoing bowel resection to poor postoperative outcomes. Furthermore, the CONUT score was found to be an independent risk factor for postoperative complications in CD. To the best of our knowledge, this was the first study to determine the correlation between the preoperative CONUT score and postoperative outcomes in CD patients. In addition, this study found that the CONUT score was better than other clinical predictors for evaluating nutritional status, such as ALB and PNI.

The CONUT score is determined by the serum albumin concentration, total cholesterol concentration, and total lymphocyte count. The concentration of serum albumin will be affected by the nutritional status and other factors, including infection, inflammatory response, and fluid retention status^19,20^. Malnutrition is common in CD, especially in active CD. Studies have reported that hypoalbuminemia is a risk factor for complications following gastrointestinal surgery^21^. Galata et al.^22^ reported that a preoperative albumin level greater than 32.6 g/L was associated with a reduced risk of complications, and hypoalbuminemia was the only independent risk factor for major postoperative complications in CD patients undergoing colorectal surgery. Preoperative nutritional optimization in CD was recommended in patients with low albumin levels to minimize postoperative complications^11^. Our results were consistent with previous findings. Our study revealed that CD patients with postoperative complications had significantly lower serum albumin concentrations.

The total peripheral lymphocyte count, one necessary component of the CONUT score, indicates the immunological status of the patient^23^. Various studies have indicated that T lymphocytes affected by the systemic inflammatory response play an important role in the depression of innate cellular immunity in cancer patients^24^. A poor prognosis in cancer patients was correlated with decreased T lymphocytes due to an inadequate immune response^20^. Neubauer et al.^25^ found the role of apoptosis of peripheral lymphocytes in intestinal inflammation in CD patients. Additionally, peripheral B1a lymphocytes were decreased in association with the severity of disease activity in CD, and B1a lymphocytes played a vital role in immune protection^26^. Thus, the total peripheral lymphocyte count might be associated with postoperative complications in CD. PNI, which includes the total peripheral lymphocyte count, has already been demonstrated to predict complications in CD; the cut-off value is less than 40^10^. Similarly, the CONUT score in our study also included the total peripheral lymphocyte count and was found to be an independent risk factor for complications in CD.

The compositional difference between the PNI and CONUT score lies in the total cholesterol concentration. It is suggested that a lower cholesterol concentration has a detrimental effect on postoperative outcomes by affecting antioxidant reserve and inflammatory response^27^. Takagi et al. reported that a low cholesterol level was associated with postoperative complications in patients who underwent gastrointestinal and hepatopancreatobiliary surgery^18^. The serum cholesterol level also plays an important role in the poor prognosis of cancer patients, because cell membrane fluidity is influenced by hypocholesterolemia, which is related to the mobility of cell surface receptors and the ability to transmit transmembrane signals^28^. Therefore, a higher CONUT score including a low cholesterol level predicted poor outcomes in CD patients undergoing surgical therapy.

Although there are some common factors between the CONUT score, PNI, and ALB, the prediction values of these three indicators are different. The ROC analyses revealed that the predictive accuracy of the CONUT score was better than that of PNI and ALB. This might be because of the higher emphasis placed on the serum albumin concentration, total cholesterol concentration, and total lymphocyte count. The combinations of these three factors enhance the utility of the CONUT score for evaluating patients’ general condition, which is consistent with previous results in cancer patients^16,20^.

The current study has several limitations to acknowledge. First, this was a retrospective observational analysis, and some residual confounding factors remained. First is the selection bias created due to the exclusive recruitment of CD patients. Second, the cutoff value needs to be evaluated in other cohorts to verify the conclusion of the current study. Last but not least, a multi-centre prospective observational study is warranted, because the outcomes might be influenced by our local experience.

In conclusion, the current study confirmed that the preoperative CONUT score predicted postoperative complications in CD patients undergoing bowel resection. The CONUT system was better than ALB and PNI at predicting postoperative complications in CD. CD patients with a CONUT score over 3.5 should be intensively monitored so that postoperative complications can be detected early.

## Materials and methods

### Patients

This study was a retrospective review of 202 CD patients undergoing surgical resection from June 2016 to June 2019 at the IBD Center, a teaching hospital of Zhejiang University. All the CD patient data were collected from patients’ medical charts in the IBD database. All CD patients were diagnosed using the accepted criteria^29^. This study was approved by the Ethics Committee of our hospital and conformed to the ethical guidelines of the 1975 Declaration of Helsinki. Written informed consent was obtained from all patients.

### Inclusion and exclusion criteria

Patients with radiologic, endoscopic, and histological diagnosis of CD according to the European Crohn’s and Colitis Organisation (ECCO) guidelines and undergoing intestinal resection due to failure of medical therapy or developed complications (structuring or penetrating) were included in this study^29^. Patients with undetermined IBD or UC, those without a surgical bowel resection, those younger than 18 years old, those with malignant disease, and those with incomplete laboratory data were excluded. Patients with emergency surgery were also excluded. Thus, 148 patients were excluded in total, 80 due to incomplete laboratory data.

### Data collection

All the baseline characteristics data, perioperative data, and laboratory data were collected from the IBD database. Baseline characteristics included age, body mass index (BMI), sex, comorbidity, smoking history, medication, and Montreal classification. CD patients exposed to preoperative infliximab were defined as having a documented dose of infliximab more than 4 weeks before surgery. For patients on preoperative corticosteroids, attempts were made to wean them to a daily dose of 5 mg prednisolone or 4 mg methylprednisolone 4 weeks before surgical intervention. Intraoperative data included operation time and surgical approach (open vs laparoscopy). Laboratory data included white blood count (WBC), red blood count (RBC), haemoglobin (Hb), total blood cholesterol, albumin (ALB), C-reactive protein (CRP), erythrocyte sedimentation rate (ESR), and lymphocyte count. The prognostic nutritional index (PNI) was calculated from the serum ALB level and total peripheral lymphocyte count (TLC), and the formula was PNI = 10 × ALB (g/dL) + 0.005 TLC (per mL)^30^. The CONUT score was calculated based on serum albumin level, peripheral lymphocyte counts, and total cholesterol concentrations as in a previous study^15^. The details of the CONUT scoring system are shown in Table 5.

**Table 5:** The evaluation of Controlling Nutritional Status (CONUT) score.

Variable	Normal	Mild	Moderate	Severe
Serum albumin (g/dL)	≥ 3.50	3.00–3.49	2.50–2.99	< 2.50
Score	0	2	4	6
Total lymphocyte count (/mm^3^)	≥ 1600	1200–1599	800–1199	< 800
Score	0	1	2	3
Total cholesterol (mg/dL)	≥ 180	140–179	100–139	< 100
Score	0	1	2	3

CONUT: controlling nutritional status.

### Definition of outcomes

The primary outcome of this study was to evaluate the predictive value of the CONUT score for postoperative complications. Postoperative complications were defined as those occurring within 30 days from the date of operation or discharge, and complications were documented using the Clavien-Dindo system^31^. A follow-up telephone interview was carried out when necessary. Mild complications included those classified grades I to II according to the Clavien-Dindo system, and major complications included those classified grades III to IV. For secondary outcomes, comparison of the prediction value between the CONUT score and other scoring systems, such as ALB and PNI, was performed.

### Statistical analysis

All of the statistical analyses were conducted using SPSS 21.0 (Armonk, NY: IBM Corp). The mean ± SD or median (range) is used to represent continuous data, while categorical data are presented as numbers (%). Student’s t test or the Mann–Whitney U test for continuous variables was performed depending on the normality of the data distribution, and the Pearson χ^2^ test or Fisher’s exact test was used to analyse the categorical variables, as appropriate. Significant associations from the univariate analyses (p < 0.05) were evaluated, and the identified independent predictors of infectious complications were then analysed by multivariate logistic regression analysis. The accuracy of predictors was assessed by receiver operating characteristic (ROC) curve analysis. A P value < 0.05 was considered to be statistically significant.

## Data Availability

The datasets generated during and/or analyzed during the current study are available from the first author or corresponding author on reasonable request.
